# Untargeted Metabolomics and Metabolite–Gene Network Analysis Predict NF-κB Inhibition in Artemisian B-Treated Triple-Negative Breast Cancer Cells

**DOI:** 10.3390/metabo16060365

**Published:** 2026-05-28

**Authors:** Shujun Shan, Ziyun Hu, Guimin Xue, Ping Yao, Peipei Du, Ruixi Gan, Junsong Wang

**Affiliations:** 1 Center of Molecular Metabolism, Nanjing University of Science and Technology, Nanjing 210094, China; ssj0902@outlook.com (S.S.); hzy@njust.edu.cn (Z.H.); 2 Department of Pharmaceutical Engineering, Jiangsu Provincial Xuzhou Pharmaceutical Vocational College, Xuzhou 221116, China; 15094348610@163.com (P.Y.); 15852171621@163.com (P.D.); 3 College of Pharmacy, Henan University of Chinese Medicine, Zhengzhou 450046, China; xueguimin123@126.com

**Keywords:** artemisian B, triple-negative breast cancer, untargeted metabolomics, NF-κB signaling, metabolic reprogramming, apoptosis, metabolite–gene network topology

## Abstract

**Highlights:**

**What are the main findings?**

- Artemisian B induces TNBC apoptosis through extensive metabolic reprogramming, which our topology-guided metabolite–gene network framework successfully maps to downstream signal transduction, predicting NF-κB as a central regulatory hub.
- This computational prediction is rigorously corroborated by parallel validation, revealing coordinated suppression of IKKα/β–IκBα–p65 phosphorylation and attenuated p65 nuclear translocation, thereby confirming the framework’s capacity to bridge metabolic perturbations with signaling outcomes.

**What are the implications of the main findings?**

- Artemisian B is positioned as a multi-target modulator that concurrently disrupts TNBC metabolic homeostasis and suppresses NF-κB-driven survival programs.
- A superior, dual-perspective analytical strategy applicable to both biochemical reaction networks and signal transduction pathways is established, enabling researchers to decode natural product pharmacology through two complementary lenses: system-level metabolic topology and targeted signaling validation.

**Abstract:**

**Background:** Triple-negative breast cancer (TNBC) remains a formidable clinical challenge due to the scarcity of targeted therapies and profound metabolic heterogeneity. Although Artemisian B, a dimeric sesquiterpene lactone derived from Artemisia argyi, exhibits potent antiproliferative activity, its comprehensive metabolic footprint and the translation of these perturbations into downstream signaling regulation remain poorly characterized. **Methods:** To address this gap, we employed an integrative analytical framework combining untargeted metabolomics, topology-guided metabolite–gene network mapping, and parallel experimental validation in MDA-MB-231 cells. This workflow systematically profiled cellular phenotypes, global metabolic reprogramming, and key signaling nodes, enabling the prioritization of high-confidence mechanistic links between metabolic alterations and signal transduction. **Results:** Artemisian B dose-dependently suppressed TNBC cell viability (IC_50_ = 12.12 μM) and triggered mitochondrial apoptosis, characterized by Bax upregulation, Bcl-2 downregulation, and caspase-9/3 activation. Untargeted metabolomics identified 129 significantly altered metabolites, reflecting extensive dysregulation across lipid peroxidation, bioenergetics, and nucleotide metabolism. Topological analysis of the metabolite–gene network identified the NF-κB pathway as a highly interconnected hub within this perturbed landscape. Parallel experimental validation corroborated this prediction, demonstrating that Artemisian B consistently suppressed the phosphorylation of IKKα/β, IκBα, and p65, while markedly attenuating p65 nuclear translocation. **Conclusions:** Artemisian B induces TNBC apoptosis through extensive metabolic reprogramming coupled with concurrent inhibition of NF-κB signaling. By seamlessly integrating untargeted metabolomics with network topology, our framework not only successfully bridges metabolic perturbations with signaling outcomes but also establishes a versatile, dual-perspective strategy applicable to both biochemical reaction networks and signal transduction pathways. This approach provides a robust predictive paradigm for decoding the multi-target pharmacological mechanisms of natural products.

## 1. Introduction

Breast cancer remains the most frequently diagnosed malignancy in women worldwide [1]. Among its molecular subtypes, triple-negative breast cancer (TNBC), characterized by the absence of estrogen receptor, progesterone receptor, and HER2 amplification, represents the most aggressive clinical phenotype with the poorest prognosis, accounting for 15–20% of all cases [2,3]. Despite initial sensitivity to conventional chemotherapy, TNBC is plagued by profound intratumoral heterogeneity and rapid acquisition of drug resistance, ultimately leading to treatment failure and underscoring an urgent need for novel therapeutic strategies [4,5]. Emerging evidence highlights that TNBC cells exhibit remarkable metabolic plasticity, frequently rewiring glycolysis, glutaminolysis, and lipid metabolism to sustain proliferation and evade cellular stress [6,7]. Consequently, targeting this metabolic vulnerability has emerged as a highly promising avenue for TNBC intervention [8].

**Figure 1:**
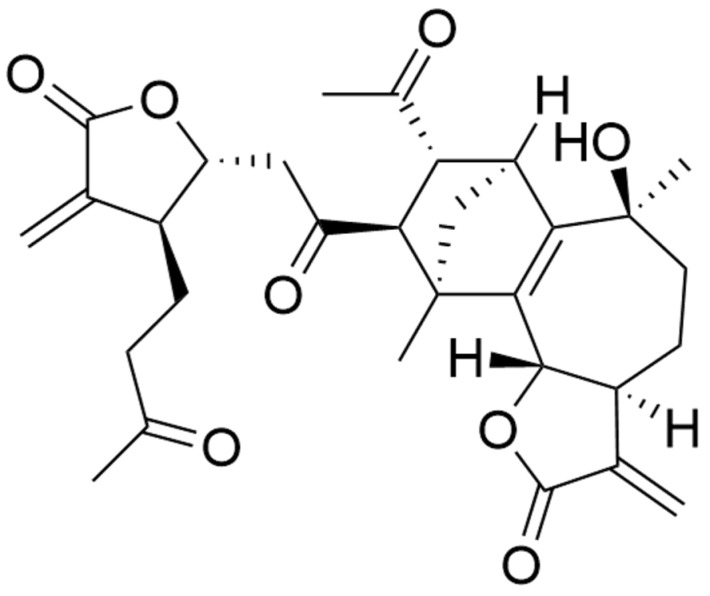
Structural formula of Artemisian B.

Natural products, distinguished by their structural complexity and inherent multi-target regulatory capacities, offer distinct advantages in modulating tumor metabolic networks [9]. Artemisian B (Figure 1), a novel dimeric sesquiterpene lactone isolated from the traditional Chinese herb *Artemisia argyi*, features a unique 1,10-4,5-diseco-guaianolide scaffold and an α-methylene-γ-lactone (αMγL) pharmacophore [10]. While preclinical studies have established its potent cytotoxicity across diverse cancer cell lines—including superior antiproliferative activity against TNBC cells via apoptosis induction and cell cycle arrest [11]—its systemic metabolic footprint and the subsequent translation of these perturbations into downstream signaling regulation remain poorly defined [12]. Current investigations have largely been confined to phenotypic endpoints, leaving a critical gap in understanding how Artemisian B-mediated metabolic reprogramming orchestrates broader signaling cascades in TNBC.

To bridge this gap, we deployed an integrative metabolomics framework recently established by our group [13], which systematically classifies drug-associated metabolites to distinguish primary pharmacological actions from secondary adaptive responses. Unlike conventional pathway enrichment analyses, this topology-guided workflow provides dual mechanistic perspectives: it simultaneously resolves system-level biochemical reaction networks and predicts downstream signal transduction hubs, thereby bridging metabolic perturbations with signaling outcomes. Originally validated in complex neuropsychiatric models, this framework demonstrates broad applicability across diverse biological contexts and disease mechanisms. Leveraging MDA-MB-231 cells as a canonical basal-like TNBC model with pronounced metabolic dependencies [14], the present study adapts this analytical strategy to elucidate the intrinsic crosstalk between Artemisian B-induced metabolic reprogramming and NF-κB signaling attenuation. By coupling computational topological prioritization with parallel experimental validation, we aim to establish a robust, predictive paradigm for decoding the multi-target mechanisms of natural products in TNBC therapy.

## 2. Materials and Methods

### 2.1. Compounds and Reagents

Artemisian B was isolated and purified from the aerial parts of *A. argyi* as previously described [10]. The structure was elucidated by comprehensive spectroscopic analyses, including nuclear magnetic resonance (^1^H/^13^C NMR) (Bruker, Karlsruhe, Germany), heteronuclear single quantum coherence (HSQC), heteronuclear multiple bond coherence (HMBC), rotating frame Overhauser effect spectroscopy (ROESY), and high-resolution electrospray ionization mass spectrometry (HRESIMS) (Agilent Technologies, Santa Clara, CA, USA). Chemical purity was confirmed to be >98% by HPLC analysis (Shimadzu, Tokyo, Japan). Prior to experiments, Artemisian B was dissolved in Dimethyl Sulfoxide (DMSO) to prepare a 100 mM stock solution and stored at −20 °C in the dark. Working solutions were prepared by diluting the stock solution in complete culture medium, ensuring a final DMSO concentration ≤ 0.1% (*v*/*v*).

The leaves of *A. argyi* were collected from Qichun County, Hubei Province, China, in May 2015, and authenticated by Professor Mian Zhang (China Pharmaceutical University) as Artemisia argyi H.Lév. & Vaniot (Asteraceae). A voucher specimen (No. 150520) has been deposited in the Herbarium of the Department of Natural Medicinal Chemistry, China Pharmaceutical University, a publicly accessible repository. *A. argyi* is neither a protected nor an endangered species, and all collection activities were conducted in accordance with institutional and local regulations.

### 2.2. Cell Culture and Treatment

Human triple-negative breast cancer MDA-MB-231 cells were purchased from the Shanghai Institute of Biochemistry and Cell Biology, Chinese Academy of Sciences (Shanghai, China). Cells were routinely cultured in Dulbecco’s Modified Eagle Medium (DMEM; Gibco Life Technologies, Billings, MT, USA) supplemented with 10% fetal bovine serum (FBS; Gibco) and 1% penicillin-streptomycin (HyClone, Marlborough, MA, USA) in a humidified incubator at 37 °C with 5% CO_2_.

For experiments, cells in the logarithmic growth phase were seeded into culture plates at an appropriate density and allowed to adhere overnight. The medium was then replaced with fresh medium containing various concentrations of Artemisian B (0, 3, 6, 12, 24, 48, 64 μM) or an equivalent volume of vehicle control (0.1% DMSO). Based on our preliminary experiments, which indicated that 48 h treatment yielded the most robust metabolic perturbations and apoptotic responses compared to 24 h, this time point was selected for subsequent metabolomics and signaling analyses. Cells were incubated for 48 h for subsequent analysis.

### 2.3. Cell Viability Assay (MTT)

MDA-MB-231 cells were seeded in 96-well plates at a density of 5 × 10^3^ cells per well and cultured for 24 h. After treatment with indicated concentrations of Artemisian B for 48 h, 20 μL of MTT solution (5 mg/mL in PBS) was added to each well, followed by incubation at 37 °C in the dark for 4 h. The medium was carefully removed, and 150 μL of DMSO was added to each well to dissolve the purple formazan crystals. Absorbance was measured at 490 nm using a microplate reader (BioTek Instruments). Cell viability was calculated relative to the control group (100%), and the half-maximal inhibitory concentration (IC_50_) was determined by nonlinear regression analysis of the dose–response curve using GraphPad Prism software (version 10.12).

### 2.4. Apoptosis Analysis by Flow Cytometry

Apoptosis was quantified using Annexin V-FITC/propidium iodide (PI) double staining. MDA-MB-231 cells were seeded in 6-well plates and treated with 12 μM Artemisian B or vehicle control for 48 h. Cells were harvested by trypsinization (including suspended cells), washed twice with ice-cold PBS, and resuspended in 1× binding buffer. Staining was performed according to the manufacturer’s protocol (Annexin V Apoptosis Detection Kit, Beyotime Biotechnology, Shanghai, China): 5 μL Annexin V-FITC and 10 μL PI were added to 100 μL cell suspension, incubated at room temperature in the dark for 30 min, followed by the addition of 400 μL binding buffer before analysis. Data were acquired on a NovoCyte flow cytometer (ACEA Biosciences, San Diego, CA, USA), and the proportions of early (Annexin V^+^/PI^−^) and late (Annexin V^+^/PI^+^) apoptotic cells were analyzed using FlowJo software (version 10.8).

### 2.5. Untargeted Metabolomics Analysis

#### 2.5.1. Sample Preparation

MDA-MB-231 cells were treated with 0, 3, 6, or 12 µM Artemisian B for 48 h (*n* = 6 per group). Metabolites were extracted on ice using 1 mL of pre-cooled methanol:water (4:1, *v*/*v*, UPLC-MS grade) per sample. After vortex mixing for 30 s and three freeze–thaw cycles (−80 °C/room temperature), proteins were precipitated at 4 °C for 1 h. Samples were then centrifuged at 16,000× *g* for 15 min at 4 °C, and the supernatants were vacuum-dried. Dried samples were reconstituted in 100 µL methanol:water (1:1, *v*/*v*), vortexed, and centrifuged at 14,000× *g* for 10 min at 4 °C. A 60 µL aliquot of the supernatant was injected for analysis.

#### 2.5.2. UHPLC-QTOF-MS Analysis

Chromatographic separation was performed on a Waters Atlantis^TM^ Premier BEH C18 AX column (2.1 mm × 100 mm, 1.7 µm) at 40 °C with a flow rate of 0.4 mL/min and an injection volume of 10 µL. Mobile phase A was 0.1% formic acid in water, and mobile phase B was acetonitrile containing 0.1% formic acid. The gradient elution program was as follows: 0–2 min, 1% B; 2–12 min, 1% → 99% B; 12–15 min, 99% B; 15–15.1 min, 99% → 1% B; 15.1–17 min, 1% B for equilibration.

Mass spectrometry was conducted on a TripleTOF 5600+ system (Sciex) equipped with an electrospray ionization (ESI) source in both positive and negative ion modes. Data were acquired in information-dependent acquisition (IDA) mode: scan range *m*/*z* 50–1200, accumulation time 250 ms; the top 10 most intense precursor ions were selected for MS/MS scanning (collision energy 35 ± 15 eV, dynamic background subtraction enabled). Ion source parameters were ion spray voltage 5500 V (positive)/4500 V (negative), source temperature 550 °C, nebulizer gas (GS1) and heater gas (GS2) 55 psi, curtain gas (CUR) 35 psi, and collision gas (CAD) medium. One pooled quality control (QC) sample was injected every five samples to monitor system stability.

#### 2.5.3. Data Processing and Metabolite Identification

Raw mass spectrometry data were converted to open format (.mzML) and processed using a standardized pipeline for peak detection, retention time alignment, and peak area integration. Metabolite annotation strictly adhered to the Metabolomics Standards Initiative (MSI) guidelines [15], categorizing identifications into three confidence levels: Level 1 (confirmed) required exact mass match (<10 ppm error), MS/MS spectral match, and co-elution with authentic standards; Level 2 (putative) relied on exact mass and MS/MS matches against public databases (HMDB, KEGG, METLIN); Level 3 (candidate) was based solely on exact mass for molecular formula assignment. To mitigate technical variability, feature intensities were normalized, and ions with >50% missing values across the dataset were excluded. A curated feature matrix containing retention time, *m*/*z*, normalized peak area, and MSI confidence level was compiled for downstream multivariate analysis (Supplementary Table S1).

#### 2.5.4. Multivariate Statistical Analysis and Differential Metabolite Screening

Unsupervised principal component analysis (PCA) was initially applied to assess overall data distribution, detect batch effects, and identify outliers. Subsequently, supervised partial least squares-discriminant analysis (PLS-DA) was employed to maximize inter-group metabolic separation. Model robustness and predictive validity were rigorously evaluated via 200 permutation tests, with thresholds of R^2^Y > 0.5 and Q^2^ > 0.3 applied to guard against overfitting. Differential metabolites were identified using a dual-filtering strategy combining multivariate and univariate metrics: variable importance in projection (VIP) > 1.0, coupled with a two-tailed Student’s *t*-test (*p* < 0.05) and an absolute fold-change threshold of |log_2_FC| > 1.

#### 2.5.5. Pathway Enrichment and Metabolite–Gene Network Topology Analysis

To isolate the core metabolic mechanisms of Artemisian B intervention, we implemented a metabolite classification and “difference-of-differences” (diff-diff) screening strategy, directly adapted from our recently established integrative metabolomics framework [16]. This approach systematically distinguishes primary pharmacological effects from secondary adaptive or compound-intrinsic responses. Significantly altered metabolites were mapped to KEGG, Reactome, HMDB, and METLIN databases. Pathway enrichment was evaluated via hypergeometric testing, with false discovery rate (FDR) correction (FDR < 0.05) applied to control for multiple comparisons.

Leveraging this classification logic, we constructed metabolite–gene interaction networks in Cytoscape v3.9.1 using KEGG RCLASS and RPAIR biochemical reaction annotations (Kanehisa Laboratories, Kyoto, Japan; https://www.kegg.jp/). Network topology analysis was performed to pinpoint high-confidence regulatory hubs: bridge metabolites were defined as nodes connecting ≥ 3 differentially expressed genes, representing potential metabolic-signaling intersections, while hub genes were identified by degree centrality within the top 20% of connectivity. The visualization and prioritization of biochemical reaction networks were guided by core pathways extracted through the diff-diff strategy (Supplementary Table S2). This topology-driven workflow systematically links metabolic perturbations to downstream signaling transduction, providing a predictive systems biology foundation for subsequent experimental validation and demonstrating the broad applicability of our classification framework beyond neuropsychiatric models to cancer pharmacology [16].

### 2.6. Immunoblot Analysis

Total cellular proteins were extracted using RIPA lysis buffer supplemented with protease and phosphatase inhibitors (Beyotime Biotechnology, Shanghai, China). Cytosolic and nuclear fractions were prepared using a Nuclear and Cytoplasmic Protein Extraction Kit (Beyotime) according to the manufacturer’s instructions. Protein concentrations were determined using a BCA Protein Assay Kit (Yeasen Biotechnology, Shanghai, China). Equal amounts of protein (30 μg) were separated by 10% SDS-PAGE and transferred onto 0.45 μm nitrocellulose membranes (Millipore, Billerica, MA, USA) by wet transfer.

Membranes were blocked with 5% non-fat milk in Tris-buffered saline containing 0.1% Tween-20 (TBST) for 2 h at room temperature, followed by overnight incubation at 4 °C with primary antibodies against GAPDH, PCNA, p65, IκBα, Bcl-2, and Bax (Proteintech, Wuhan, China; 1:1000), or caspase-3, cleaved caspase-3, caspase-9, cleaved caspase-9, p-p65 (Ser536), p-IκBα (Ser32), IKKα/β, and p-IKKα/β (Ser176/180) (Affinity Biosciences, Changzhou, China; 1:1000). After three washes with TBST, membranes were incubated with HRP-conjugated goat anti-mouse or goat anti-rabbit secondary antibodies (Proteintech; 1:5000) for 1 h at room temperature. Signals were visualized using an enhanced chemiluminescence (ECL) substrate (Yeasen) and captured on a ChemiScope 6100 imaging system (Clinx Science Instruments, Shanghai, China). Band intensities were quantified using ImageJ software (version 1.53; NIH, New York, NY, USA) and normalized to GAPDH for cytosolic/total proteins or PCNA for nuclear proteins.

### 2.7. Statistical Analysis

All quantitative data are presented as mean ± standard deviation (SD) from at least three independent biological replicates. Statistical analyses were performed using GraphPad Prism software (version 10.12; GraphPad Software, San Diego, CA, USA) or R software (version 4.2.1; R Foundation for Statistical Computing, Vienna, Austria). For comparisons among multiple groups, normality and homogeneity of variance were first assessed using the Shapiro–Wilk test and Levene’s test, respectively. Data meeting these assumptions were analyzed by one-way analysis of variance (ANOVA) followed by Tukey’s post hoc test; data failing to meet the assumptions were subjected to data transformation or analyzed using the Kruskal–Wallis nonparametric test. Comparisons between two groups were conducted using two-tailed Student’s *t*-test. For pathway enrichment analysis in metabolomics, raw *p*-values were corrected for multiple testing using the Benjamini–Hochberg method to control the FDR < 0.05. A *p* < 0.05 was considered statistically significant.

## 3. Results

### 3.1. Artemisian B Reduces Cell Viability and Induces Morphological Alterations in MDA-MB-231 Cells

**Figure 2:**
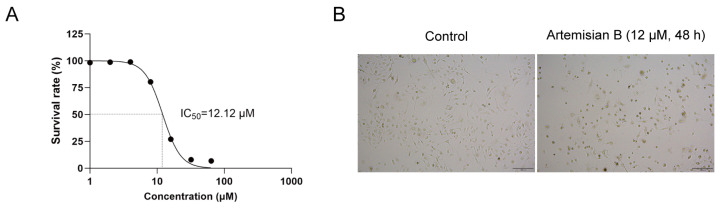
Dose-dependent reduction in cell viability and morphological alterations induced by Artemisian B in MDA-MB-231 cells. (**A**) Cell viability was assessed by MTT assay after 48 h treatment with varying concentrations of Artemisian B (0, 1, 2, 4, 8, 16, 32, 64 μM). The half-maximal inhibitory concentration (IC_50_) was calculated by nonlinear regression analysis. (**B**) Representative morphological changes in MDA-MB-231 cells after 48 h treatment with Artemisian B, as observed under an inverted microscope. Control cells show normal adherent morphology, while treated cells exhibit rounding, shrinkage, and detachment. Scale bar = 200 μm.

The MTT assay showed that treatment with Artemisian B for 48 h resulted in a dose-dependent reduction in the viability of MDA-MB-231 cells (Figure 2A). Within the tested concentration range (0–64 μM), cell viability decreased from 100% in the control group to (6.78 ± 0.2)% in the 64 μM group, with a calculated half-maximal inhibitory concentration (IC_50_) of 12.12 μM. Inverted microscopy revealed that, compared to the normal adherent morphology of control cells, MDA-MB-231 cells treated with 12.12 μM Artemisian B for 48 h exhibited significant morphological alterations, including cell rounding, shrinkage, and decreased adherence (Figure 2B).

### 3.2. Artemisian B Induces Apoptosis in MDA-MB-231 Cells

**Figure 3:**
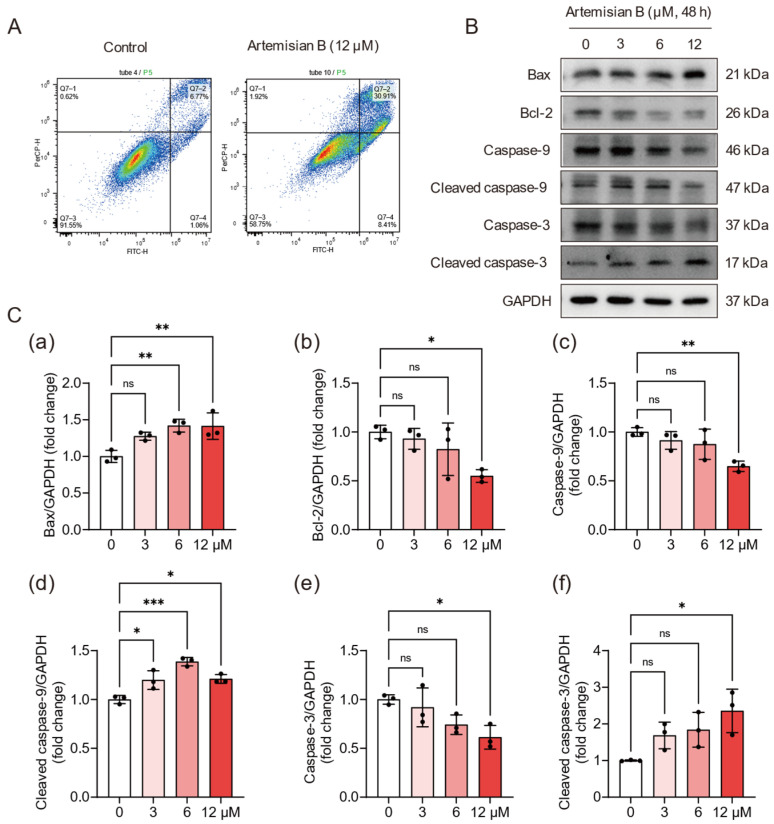
Artemisian B induces apoptosis in MDA-MB-231 cells. (**A**) Representative flow cytometry dot plots of Annexin V-FITC/PI staining after treatment with 12 μM Artemisian B for 48 h. Percentages in quadrants represent early (lower right, Annexin V^+^/PI^−^) and late (upper right, Annexin V^+^/PI^+^) apoptotic cells. The color gradient represents cell density, ranging from blue (low) to red (high). (**B**) Western blot analysis of apoptosis-related proteins, including Bax, Bcl-2, caspase-9, cleaved caspase-9, caspase-3, and cleaved caspase-3. Cells were treated with indicated concentrations (0, 3, 6, 12 μM) for 48 h. GAPDH served as a loading control. (**C**) Quantitative analysis of protein expression levels normalized to GAPDH. Data are presented as mean ± SD (*n* = 3). * *p* < 0.05, ** *p* < 0.01, *** *p* < 0.001 vs. control group; ns, not significant.

Annexin V-FITC/PI double staining flow cytometry analysis showed that treatment with 12 μM Artemisian B for 48 h significantly increased the total apoptotic cell population from 2.98% in the control group to 39.31% (Figure 3A). This included 8.41% early apoptotic cells (Annexin V^+^/PI^−^) and 30.90% late apoptotic/necrotic cells (Annexin V^+^/PI^+^). Western blot analysis further demonstrated significant alterations in the expression of key apoptosis-related proteins following Artemisian B treatment (Figure 3B,C). The expression level of the pro-apoptotic protein Bax was significantly increased to 1.38-fold (** *p* < 0.01) and 1.35-fold (** *p* < 0.01) in the 6 μM and 12 μM groups, respectively, compared to the control group. Conversely, the expression of the anti-apoptotic protein Bcl-2 decreased to 0.56-fold in the 12 μM group (* *p* < 0.05). Consistent with caspase cascade activation, Cleaved caspase-9 levels were elevated at 3 μM (* *p* < 0.05) group and peaked at 6 μM (*** *p* < 0.001), remaining significantly higher than control at 12 μM (* *p* < 0.05). Similarly, Cleaved caspase-3 levels significantly increased to 2.41-fold (* *p* < 0.05) in the 12 μM group. Concurrently, the levels of full-length caspase-9 and caspase-3 proteins decreased in a dose-dependent manner. Collectively, these findings characterize the apoptotic phenotype induced by Artemisian B, which is accompanied by a shift in the Bax/Bcl-2 ratio and concurrent activation of the caspase cascade.

### 3.3. Artemisian B Elicits Dose-Dependent Metabolic Perturbations in TNBC Cells

**Figure 4:**
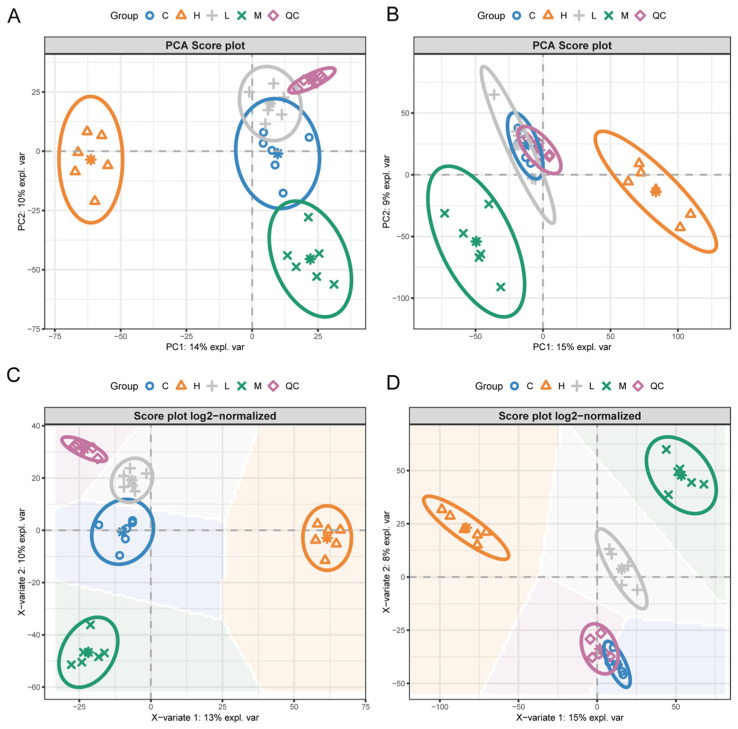
Multivariate analysis reveals dose-dependent metabolic profile alterations induced by Artemisian B. (**A**,**B**) PCA score plots derived from data acquired in negative (**A**) and positive (**B**) ionization modes. (**C**,**D**) PLS-DA score plots derived from data acquired in negative (**C**) and positive (**D**) ionization modes. Groups are indicated by distinct symbols and colors: Control (C, blue circle), Low dose (L, 3 μM, orange triangle), Medium dose (M, 6 μM, gray cross), High dose (H, 12 μM, pink diamond), and Quality Control (QC, green cross). Ellipses represent 95% confidence intervals. The shaded background regions in panels C and D indicate the PLS-DA classification boundaries (predictive zones) for each group. Principal components (PC) and latent variables (X-variates) are labeled with their respective explained variance percentages.

**Figure 5:**
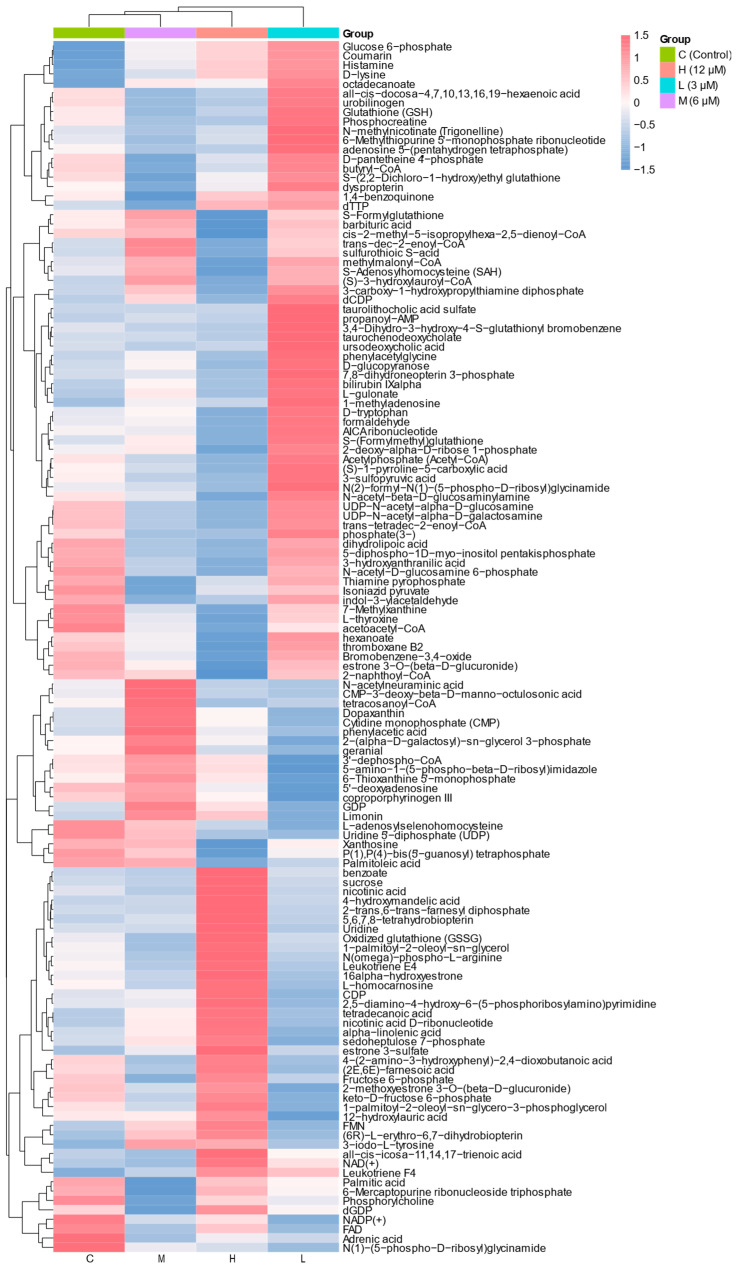
Hierarchical clustering heatmap visualizing dose-dependent metabolic perturbations. The heatmap displays the Z-scored relative abundance of 129 significantly altered metabolites across Control (C), Low (L, 3 μM), Medium (M, 6 μM), and High (H, 12 μM) dose groups. Columns represent biological replicates, ordered by hierarchical clustering based on metabolite abundance similarity. Red and blue gradients indicate up- and down-regulation, respectively. Metabolites were filtered based on VIP > 1, and |log_2_FC| > 1.

Multivariate statistical analysis revealed distinct metabolic profile shifts between Artemisian B-treated and control groups. PCA score plots demonstrated clear separation of treatment groups from controls in both negative and positive ionization modes (Figure 4A,B), while the tight clustering of QC samples confirmed the high stability and reproducibility of the acquisition system. Supervised PLS-DA further resolved a concentration-dependent trajectory, with samples from low- (3 μM), medium- (6 μM), and high-dose (12 μM) groups exhibiting progressive displacement from the control cluster along the primary latent variables (Figure 4C,D). Collectively, these multivariate patterns indicate that Artemisian B systematically alters the metabolic homeostasis of MDA-MB-231 cells in a dose-responsive manner. Untargeted profiling identified 144 endogenous metabolites spanning lipids, amino acids, nucleotides, carbohydrates, and cofactors (Table S1). Application of combined multivariate (VIP > 1) and univariate (* *p* < 0.05, |log_2_FC| > 1) filtering criteria yielded 129 significantly altered metabolites (89.6% of identified features) across at least one dose group (Figure 5), reflecting extensive drug-associated metabolic perturbation. Hierarchical clustering of these differential metabolites revealed distinct fingerprint signatures, with clear segregation among dose levels. Notably, response pattern analysis classified the 129 features into monotonic (62, 48.1%), threshold (35, 27.1%), and biphasic (17, 13.2%) trajectories. These kinetic profiles establish a robust, concentration-dependent metabolic baseline, which serves as the foundational dataset for subsequent metabolite–gene network topology prediction and parallel molecular validation.

### 3.4. Identification of Hub Metabolites and Enrichment Analysis of Core Signaling Pathways

**Figure 6:**
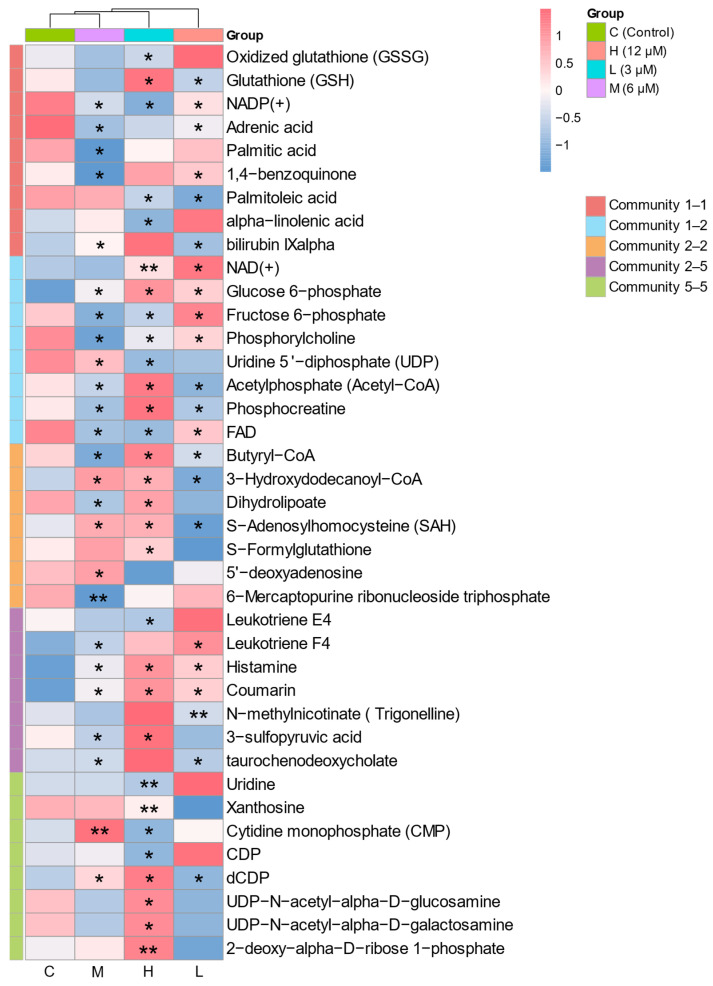
Dose-dependent abundance patterns and functional module distribution of hub metabolites. The heatmap displays the Z-scored relative abundance of 39 empirically detected hub metabolites (each connected to ≥3 co-annotated genes) across dose groups. Columns represent biological replicates ordered by hierarchical clustering. Rows are grouped by functional communities indicated by the left color bar. The color scale indicates Z-scored relative abundance. Asterisks denote statistical significance versus control (* *p* < 0.05, ** *p* < 0.01).

**Figure 7:**
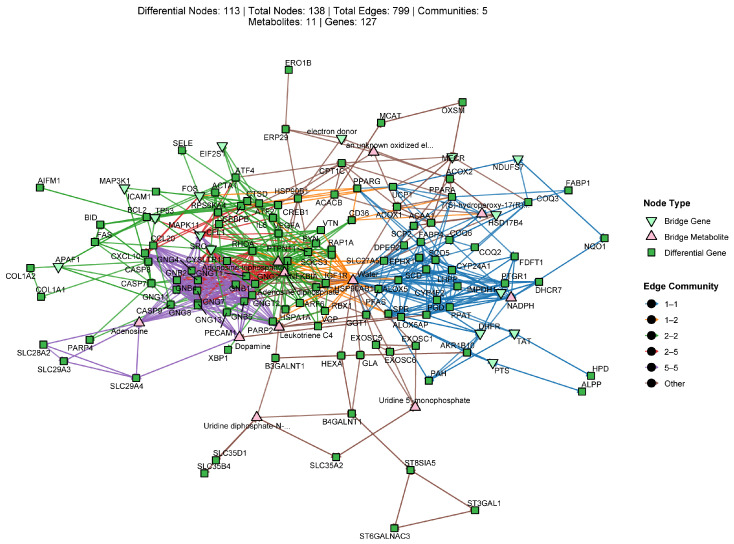
Metabolite–gene association network derived from pathway database mapping. The network comprises 138 nodes (11 core metabolites, 127 genes) and 799 edges constructed based on KEGG RCLASS and RPAIR biochemical annotations. Nodes are shaped by type and colored by functional community. Edge colors represent community membership. The network topologically highlights the Apoptosis and Survival Signaling (2–2) and Kinase and Transcriptional Regulation (2–5) communities as highly connected modules.

To systematically map the associations between differential metabolites and co-annotated genes, we constructed a metabolite–gene association network using the 144 identified endogenous metabolites. Edges in the network denote database-inferred biochemical linkages based on KEGG RCLASS and RPAIR annotations, reflecting potential pathway co-occurrence. Modular analysis using the Louvain algorithm partitioned the network into seven functional communities, each enriched for specific biological themes: Lipid Metabolism and Oxidative Stress (1–1), Metabolic-Signaling Crosstalk (1–2), Apoptosis and Survival Signaling (2–2), Kinase and Transcriptional Regulation (2–5), GPCR Signaling and Inflammatory Mediators (5–5), Peroxisomal Fatty Acid Oxidation (4–4), and Glycosylation and Nucleotide Transport (3–3). To prioritize high-confidence nodes, we applied a dual-filtering strategy. First, topological analysis identified 11 theoretical bridge metabolites that connect multiple co-annotated genes within pathway databases, representing potential metabolic-signaling intersections. Second, integrating this with experimental data, we pinpointed 39 empirically detected hub metabolites that were significantly altered (VIP > 1, *p* < 0.05) in at least one dose group and linked to ≥3 co-annotated genes. A hierarchical clustering heatmap visualized the dose-dependent abundance patterns of these hub metabolites across groups (Figure 6). This analysis revealed a broad functional distribution, encompassing redox homeostasis (GSSG/GSH), energy metabolism (NAD^+^/Phosphocreatine), inflammatory signaling (Leukotriene F4/Histamine), and nucleotide metabolism (Uridine/Xanthosine). Notably, certain theoretical bridge metabolites (e.g., NADPH, ATP, Leukotriene C4) were not directly quantified, likely due to detection limits or ionization properties; however, their functionally related, empirically detected counterparts exhibited significant dose-dependent shifts, collectively highlighting these core processes. Subsequently, applying a “difference-of-differences” (diff-diff) screening strategy, significantly altered metabolites were mapped to KEGG and Reactome databases. Enrichment significance was evaluated via hypergeometric testing (FDR < 0.05) to rank core pathways. Using Cytoscape v3.9.1, we generated a visualized association network comprising 138 nodes (11 core metabolites, 127 genes) and 799 edges (Figure 7). Topological assessment indicated the highest node connectivity within the Apoptosis and Survival Signaling (2–2) and Kinase and Transcriptional Regulation (2–5) communities. Notably, these modules displayed the greatest number of edges linking to NF-κB pathway-related genes (RELA, NFKB1, IKBKB). Collectively, these topological features predict the NF-κB pathway as a highly interconnected node within the altered metabolic landscape, providing a prioritized target for parallel experimental validation.

### 3.5. Parallel Experimental Characterization of NF-κB Pathway Alterations

**Figure 8:**
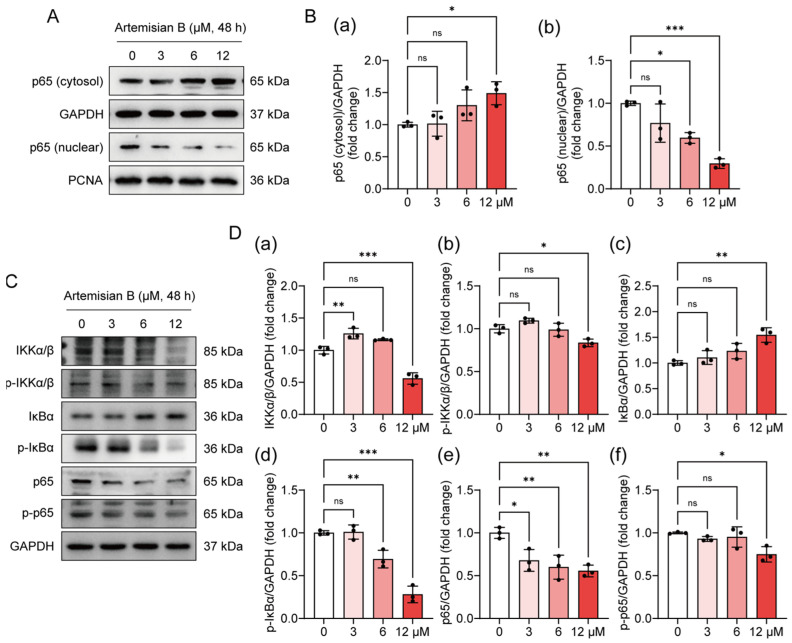
Artemisian B suppresses the NF-κB signaling pathway in MDA-MB-231 cells. (**A**,**B**) Western blot analysis and quantification of p65 protein expression in nuclear and cytoplasmic extracts after 48 h treatment with indicated concentrations of Artemisian B (0, 3, 6, 12 μM). PCNA and GAPDH served as loading controls for nuclear and cytoplasmic fractions, respectively. (**C**,**D**) Western blot analysis and quantification of expression and phosphorylation levels of key NF-κB pathway proteins (IKKα/β, p-IKKα/β, IκBα, p-IκBα, p65, p-p65) after 48 h treatment with indicated concentrations of Artemisian B. GAPDH was used as a loading control for total protein. Data are presented as mean ± SD from three independent experiments. * *p* < 0.05, ** *p* < 0.01, *** *p* < 0.001; ns, not significant.

To experimentally assess the status of the NF-κB pathway prioritized by the metabolite–gene network analysis in Section 3.4, we examined the expression, phosphorylation, and subcellular localization of key pathway proteins. Nuclear-cytoplasmic fractionation assays revealed a concentration-dependent increase in cytoplasmic p65 protein levels, with a statistically significant elevation at 12 μM (Figure 8A,B(a)). Conversely, nuclear p65 levels were significantly reduced at 6 μM and 12 μM Figure 8A,B(b)). These observations indicate that Artemisian B treatment is associated with attenuated p65 nuclear translocation, limiting its availability for downstream transcriptional activation. Parallel assessment of upstream regulatory nodes showed that Artemisian B exposure significantly reduced the phosphorylation levels of the IKK complex and IκBα. Specifically, p-IKKα/β levels were significantly decreased at 12 μM (Figure 8C,D(b)), while p-IκBα levels were significantly lowered at both 6 μM and 12 μM (Figure 8C,D(d)). Notably, total IκBα protein accumulated significantly at 12 μM (Figure 8C,D(c)), a pattern consistent with reduced phosphorylation-dependent degradation and consequent cytoplasmic retention. Concurrently, total p65 protein exhibited a dose-dependent decline across treatment groups (Figure 8C,D(e)), and its phosphorylation level (p-p65) was significantly suppressed at 12 μM (Figure 8C,D(f)). Collectively, these molecular observations indicate a dose-dependent attenuation of NF-κB signaling activity, characterized by altered kinase phosphorylation status, IκBα stabilization, and restricted p65 nuclear import. These parallel experimental findings align with the topological prediction of NF-κB suppression derived from network analysis and coincide with the apoptotic phenotype documented in Section 3.2.

## 4. Discussion

This study employed a discovery-driven workflow integrating untargeted metabolomics, database-informed metabolite–gene network mapping, and parallel molecular characterization to systematically profile the multi-level biological responses of MDA-MB-231 cells to Artemisian B intervention. Untargeted profiling revealed extensive, dose-dependent metabolic perturbations spanning redox homeostasis, bioenergetics, and nucleotide pools. Topological analysis of the KEGG-annotated association network prioritized the NF-κB pathway as a highly interconnected node within this altered metabolic landscape. Parallel biochemical validation confirmed a concomitant attenuation of NF-κB signaling, characterized by reduced phosphorylation of IKKα/β, IκBα, and p65, alongside restricted p65 nuclear translocation. These findings align with the apoptotic phenotype documented in Section 3.2 and demonstrate the predictive utility of integrating untargeted metabolomics with network topology to map the multi-target pharmacological profiles of natural products. By simultaneously resolving system-level biochemical reaction networks and pinpointing downstream signal transduction hubs, this dual-perspective framework bridges metabolic perturbations with signaling outcomes. The following discussion interprets these observations through the lens of functional module co-occurrence, topological prioritization, and established biochemical paradigms, while explicitly delineating the hypothesis-generating nature of network-derived inferences.

### 4.1. Co-Occurrence of Lipid Metabolism Perturbations and Redox Imbalance

The metabolite–gene association network partitioned lipid metabolism and oxidative stress into a distinct functional community (1–1). Empirical profiling demonstrated a pronounced accumulation of oxidized glutathione (GSSG) in the high-dose group, whereas reduced glutathione (GSH) exhibited a transient compensatory elevation at intermediate doses before declining. This dynamic trajectory indicates a progressive shift toward oxidative stress, suggesting that the cellular redox buffering capacity was increasingly challenged under sustained Artemisian B exposure. Topological mapping identified NADPH as a bridge metabolite linking multiple co-annotated genes (e.g., PTGR1, DHCR7, ALOX5AP), reflecting database-inferred biochemical proximity rather than direct regulatory control. Concurrently, the depletion of adrenic acid (low/medium doses) and palmitic acid (medium dose), coupled with community-level enrichment of peroxisomal fatty acid oxidation (4–4), points to a broad remodeling of long-chain fatty acid handling [17,18]. It is important to note that network community assignment denotes functional co-occurrence rather than hierarchical dominance; key enzymatic nodes such as ACOX1 and CPT1C were distributed across overlapping modules, underscoring the inherent limitations of static database topology in resolving context-dependent metabolic crosstalk [19,20,21]. Consequently, our interpretation prioritizes empirically quantified metabolite shifts over topological centrality. These coordinated alterations are consistent with established paradigms linking reductive power depletion to compromised lipid peroxidation defense and membrane remodeling, precluding definitive causal assignment between redox disruption and fatty acid metabolism; the robust co-occurrence pattern establishes a clear phenotypic baseline for future targeted flux or isotopic tracing studies.

### 4.2. Concurrent Manifestation of Energetic Shifts and Apoptotic Execution

Within the apoptosis and survival signaling community (2–2), phosphocreatine exhibited a biphasic trajectory: transient elevation at low dose followed by marked depletion at higher concentrations. Concurrently, NAD^+^ accumulation and FAD reduction suggest altered mitochondrial redox coupling efficiency. These energetic fluctuations manifested in synchrony with canonical apoptotic markers, including Bax upregulation, Bcl-2 downregulation, and caspase-9/3 cleavage [8,22]. Topological mapping positioned ATP as a bridge node connecting to caspase-9 within KEGG reaction annotations, reflecting the well-documented biochemical requirement for ATP in apoptosome assembly [23]. The observed metabolic shifts likely correlate with the energetic prerequisites for caspase activation, though direct mechanistic linkage would require kinetic or metabolic intervention assays [24]. Notably, literature evidence indicates that energy-sensing kinases (e.g., AMPK) can modulate IKK/NF-κB activity under metabolic stress, providing a plausible theoretical interface between bioenergetic status and inflammatory signaling [25]. Importantly, the present data characterize these phenomena as concurrent hallmarks of Artemisian B intervention rather than a validated metabolic-to-signaling causal cascade. This distinction reinforces the framework’s capacity to map parallel biological processes, enabling researchers to identify functionally aligned modules before committing to targeted mechanistic dissection.

### 4.3. Topological Prediction and Parallel Validation of NF-κB Pathway Attenuation

Network topology prioritized communities 1–2 (metabolic-signaling crosstalk) and 2–5 (kinase and transcriptional regulation) as highly interconnected modules. Within this architecture, NFKBIA (IκBα) emerged as a high-centrality node, with database-mapped edges linking it to ATP turnover and SRC kinase annotations. While KEGG-based network construction reflects co-enrichment and potential biochemical proximity rather than direct physical interaction, these topological features align precisely with canonical IKK/IκBα/p65 regulatory paradigms: attenuated kinase activity or energetic constraints can limit IκBα phosphorylation, thereby stabilizing the inhibitory complex and sequestering NF-κB (p65) in the cytoplasm [26]. Parallel experimental characterization directly corroborated this topological prediction, demonstrating dose-dependent suppression of IKKα/β, IκBα, and p65 phosphorylation, alongside significantly reduced nuclear p65 accumulation. The biological significance of NF-κB attenuation is well established in the context of survival-associated transcriptional programming [27,28]. Although network topology alone cannot dictate transcriptional directionality, the co-clustering of BCL2 and IL6 within survival modules is consistent with extensive documentation of NF-κB-dependent anti-apoptotic and pro-inflammatory gene activation [29]. The observed stabilization of IκBα and restricted p65 nuclear import therefore aligns with diminished output from these survival nodes, coinciding with the dose-dependent escalation of apoptotic execution [30]. Furthermore, community 2–5 harbored a distinct cluster of stress-responsive transcription factors (MAPK11/p38, CREB1, ATF2). Metabolomic profiling revealed elevated histamine and coumarin levels at intermediate/high doses, metabolites known to engage GPCR-mediated cascades under metabolic stress [27,29]. However, topological metrics (degree and betweenness centrality) for these nodes were comparatively peripheral, suggesting that associated inflammatory or stress signals likely represent parallel adaptive responses rather than primary drivers of the cytotoxic phenotype. This hierarchical prioritization exemplifies how topology-guided screening can effectively separate core mechanistic targets from secondary compensatory pathways [31].

### 4.4. Nucleotide Pool Imbalance and Putative Epigenetic-Glycosylation Crosstalk

Untargeted profiling revealed pronounced alterations in nucleotide and one-carbon metabolism. High-dose treatment induced significant uridine accumulation alongside xanthosine depletion, while CMP and dCDP exhibited dose-dependent elevations. Concurrently, S-adenosylhomocysteine (SAH) levels were markedly reduced in the high-dose group. Network mapping associated these shifts with communities 3–3 (glycosylation and nucleotide transport) and 5–5 (GPCR and inflammatory mediators). Community 3–3 was enriched for SLC35 nucleotide-sugar transporters and EXOSC complex nodes, whereas community 5–5 displayed lower topological centrality, consistent with its likely role as a secondary response module. Interpreting these findings through established biochemical frameworks, purine–pyrimidine pool imbalances may compromise nucleic acid synthesis and DNA damage repair capacity, potentially reducing proliferative resilience [32]. The functional dependence of SLC35 transporters and EXOSC machinery on UDP-sugar donors suggests that energetic and redox constraints could indirectly limit protein N- and O-linked glycosylation, a post-translational modification known to influence membrane receptor stability and extracellular signal sensing [33]. At the epigenetic level, the decline in SAH—a competitive inhibitor of histone and DNA methyltransferases—implies perturbation of the SAM/SAH axis, which may influence activating histone marks (e.g., H3K4me3) and contribute to the transcriptional silencing of survival-associated genes [34,35]. Collectively, these nucleotide, glycosylation, and one-carbon metabolic features exhibit topological co-association within the network. While the present study characterizes these as concurrent metabolic signatures rather than causally linked events, their integration forms a coherent, hypothesis-generating framework. Future targeted metabolite tracing, glycoproteomic profiling, and chromatin accessibility assays will be required to dissect the precise molecular interfaces underlying this multi-dimensional metabolic response [36].

## 5. Conclusions

This study demonstrates that Artemisian B exerts dose-dependent antiproliferative and pro-apoptotic effects in TNBC cells, coinciding with extensive metabolic reprogramming across redox, bioenergetic, and nucleotide pools. By integrating untargeted metabolomics with topology-guided metabolite–gene network mapping, we successfully prioritized the NF-κB pathway as a central regulatory hub within this perturbed landscape. Parallel experimental validation corroborated this topological prediction, revealing coordinated suppression of IKKα/β–IκBα–p65 phosphorylation and attenuated p65 nuclear translocation. Collectively, these findings characterize Artemisian B as a multi-target modulator that concurrently disrupts metabolic homeostasis and dampens NF-κB-driven survival signaling.

Importantly, the present workflow establishes a robust predictive framework that bridges system-level metabolic topology with targeted signaling validation. Rather than asserting direct mechanistic causality between specific metabolic shifts and downstream kinase activation, this dual-perspective strategy effectively separates high-confidence regulatory hubs from secondary adaptive responses. By simultaneously resolving biochemical reaction networks and pinpointing signal transduction pathways, the approach offers a versatile analytical paradigm for decoding the complex pharmacological profiles of natural products.

While the unique 1,10-4,5-diseco-guaianolide scaffold of Artemisian B warrants further target deconvolution and structure–activity relationship studies, the current correlative evidence provides a strong foundation for mechanistic follow-up. Future investigations employing genetic perturbation, isotopic metabolic flux tracing, and rescue assays will be essential to delineate precise causal interfaces between metabolic nodes and NF-κB regulation. Concurrently, comprehensive in vivo pharmacokinetic profiling and evaluation of combinatorial regimens with established TNBC therapeutics (e.g., PARP inhibitors or immune checkpoint modulators) will be critical for advancing Artemisian B toward clinical translation.

## Data Availability

The data presented in this study are available within the article/Supplementary Material. Additional inquiries can be directed to the corresponding authors.

## Supplementary Materials

The following supporting information can be downloaded at: https://www.mdpi.com/article/10.3390/metabo16060365/s1, Table S1: Relative abundance and network topological parameters of differential metabolites identified by untargeted metabolomics in MDA-MB-231 cells treated with Artemisian B; Table S2: Expression profiles and functional annotation of differential genes connected to hub metabolites in the metabolite–gene regulatory network.

## Abbreviations

The following abbreviations are used in this manuscript:

diff-diff	difference-of-differences
DMEM	Dissolved In Dimethyl Sulfoxide
DMSO	Dissolved In Dimethyl Sulfoxide
ESI	electrospray ionization
FDR	false discovery rate
HMBC	heteronuclear multiple bond coherence
HRESIMS	high-resolution electrospray ionization mass spectrometry
HSQC	heteronuclear single quantum coherence
IDA	information-dependent acquisition
MSI	metabolomics standards initiative
NMR	nuclear magnetic resonance
PCA	principal component analysis
PI	propidium iodide
PLS-DA	partial least squares-discriminant analysis
QC	quality control
ROESY	rotating frame Overhauser effect spectroscopy
SAH	S-adenosylhomocysteine
SAM	S-adenosylmethionine
SD	standard deviation
VIP	variable importance in projection
TNBC	triple-negative breast cancer
